# EWELD: A Large-Scale Industrial and Commercial Load Dataset in Extreme Weather Events

**DOI:** 10.1038/s41597-023-02503-6

**Published:** 2023-09-11

**Authors:** Guolong Liu, Jinjie Liu, Yan Bai, Chengwei Wang, Haosheng Wang, Huan Zhao, Gaoqi Liang, Junhua Zhao, Jing Qiu

**Affiliations:** 1 School of Science and Engineering, The Chinese University of Hong Kong, Shenzhen, 518172 China; 2 Shenzhen Institute of Artificial Intelligence and Robotics for Society, Shenzhen, 518129 China; 3 School of Data Science, The Chinese University of Hong Kong, Shenzhen, 518172 China; 4 School of Mechanical Engineering and Automation, Harbin Institute of Technology, Shenzhen, Shenzhen, 518055 China; 5 School of Electrical and Information Engineering, The University of Sydney, Sydney, NSW 2006 Australia

**Keywords:** Energy modelling, Electrical and electronic engineering

## Abstract

Load forecasting is crucial for the economic and secure operation of power systems. Extreme weather events, such as extreme heat and typhoons, can lead to more significant fluctuations in power consumption, making load forecasting more difficult. At present, due to the lack of relevant public data, the research on load forecasting under extreme weather events is still blank, so it is necessary to release a large-scale load dataset containing extreme weather events. The dataset includes electricity consumption data of industrial and commercial users under extreme weather events such as typhoons and extreme heat, which are collected at 15-minute intervals. The data is collected over six years from smart meters installed at the power entry points of users in southern China. The dataset consists of electricity consumption data from 386 industrial and commercial users in 17 industries, with more than 50 million records. During the recording period, extreme weather events such as typhoons and extreme heat are marked to form a total of 5,741 event records.

## Background & Summary

With the increasing penetration of distributed  energy resources (DER), the need for load forecasting at all levels of the distribution system has increased dramatically^1^. Underestimating electricity demand can lead to low-quality service (even blackouts) provided by utility companies and pose a severe threat to the safe and stable operation of the power grid. Therefore, accurate short-term load forecasting is the basis of power system operation and planning, supporting many services such as:

- unit commitment and scheduling of maintenance, i.e., electricity utility companies make unit commitment decisions and device maintenance plans based on the result of load forecasting^2,3^;
- demand-side management, i.e., electricity market participants can adjust their bidding strategies based on demand-side management models supported by load forecasting^4^;
- cogeneration, i.e., planning and optimizing electricity and heat production in combined heat and power plants based on the forecasting results to increase economic efficiency^5^;
- system stability and security, accurate forecast results can help the grid balance generation and demand, thus ensuring system stability and security^6–8^.

Currently, there are some publicly available load datasets that provide such data at different scales and details for the above services. Most of these datasets contain appliance power consumption data of residential users. For example, the REFIT^9^ electrical load measurement dataset consists of eight-second interval electrical measurements of the total load of the entire house, collected continuously from 20 houses over two years. In BLUED^10^, voltage and current measurements of a US residence are sampled at 12 kHz for a week. Load data is recorded in UK-DALE^11^ for five residential users with a sampling rate of 16 kHz, one of which is recorded for 655 days. There are few non-residential load datasets, and they are mainly medium voltage or higher voltage load data^12^. ISO provides utility-scale load data for a total of 46 months from March 1, 2003, to December 31, 2006 in New Zealand^13^. In particular, since core information such as sales and operating conditions of a company can be analyzed from its load data, such kind of data is regarded as a commercial secret and is rarely disclosed^14^. To the best of the authors’ knowledge, public industrial load datasets are limited. A one-year dataset of electricity load curves with a temporal resolution of 15 minutes for 50 small and mid-size enterprises in Germany is released^15^. The electricity data of 10 manufacturing companies is collected at one-minute intervals for seven months from 1 March to 30 September 2019^14^. The electricity consumption data of food and paper industries is presented^16^ and machine-level load data of a paper manufacturing factory in Brazil is investigated^17^.

To make matters worse, load forecasting is more difficult because extreme weather events can greatly affect inherent electricity usage patterns, such as extremely hot weather, which may lead to increased electricity consumption. Moreover, there are almost no relevant studies and public data on load forecasting under extreme weather events, which further increases the difficulty of load forecasting under extreme weather conditions. To solve the above problems, load data including large-scale industrial and commercial users under extreme weather events is collected and formed a dataset named Extreme Weather Events Load Dataset (EWELD) in this paper. EWELD is a public load dataset consisting of electricity consumption data from different industrial and commercial users with a sampling interval of 15 minutes. The electricity consumption data of 386 companies in 17 industries over a period of six years is recorded in this dataset, extreme weather events such as typhoons and extreme heat in the corresponding period are also recorded. The main contributions of this paper are as follows:

- The load data of 386 enterprises in 17 industries for more than six years is collected and released. Extreme weather events during the load data collection time are collected and analyzed.
- Electricity consumption patterns under extreme weather events and those under non-extreme weather events are compared and analyzed.
- All data is provided in a unified and structured format with no missing or abnormal data and is easy to use.

## Methods

In this section, methods used to create the Extreme Weather Events Load Dataset (EWELD) are introduced. First, the communication platform for collecting meteorological data and users’ electricity consumption data is described. Second, a measurement campaign conducted in large-scale industrial and commercial users in South China is presented. Finally, the processing method of the collected data and the generation method of the dataset are introduced in detail. The data in this dataset comes from an industrial partner, a power retailer with many industrial and commercial customers operating mainly in South China. This dataset focuses solely on commercial and industrial users and does not include residential data. The dataset contains data from industrial and commercial users in 17 different industries. Before data collection, each business and industry user in the dataset was informed of the data collection procedures and gave their consent. Furthermore, to protect the privacy of these users, we have implemented strict anonymization measures to ensure the identification of users cannot be identified from the data. These anonymization measures include data masking, redaction, and no direct identifiers. For data masking, the identifiable data, such as a user’s name, address, or other direct identifiers, are replaced by encoded references. These coded references do not correlate with the original data and cannot be reverse-engineered. For redaction, specific data values, especially outliers that might identify a specific user, are redacted or modified for consistency and anonymity. For no direct identifiers, direct identifiers such as phone numbers, addresses, or specific geographical locations, are omitted or transformed when forming the dataset. By employing these anonymization procedures, we aim to strike a balance between data utility and privacy, ensuring that the data is useful for research without compromising the identity and confidentiality of the users involved.

### The communication platform

**Fig. 1:**
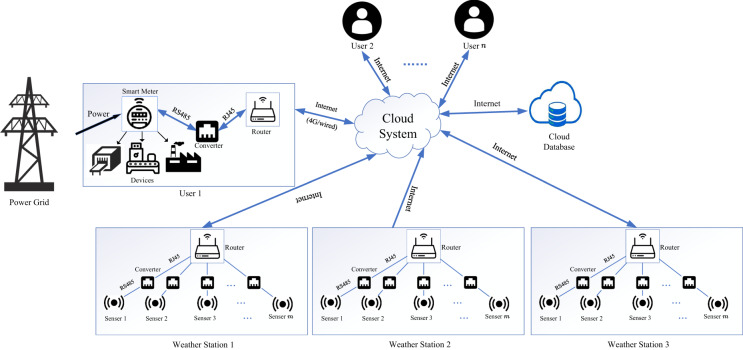
The proposed communication platform.

In the proposed communication platform, there are four main components: users, weather stations, the cloud system and the cloud database. The overall communication platform is illustrated in Fig. 1. For users, power grids provide them with electricity to supply the devices they belong to. The electricity consumption of the user is measured by the smart meter installed at the power entry point of the user. From December 2015 to April 2018, smart meters were gradually installed in multiple industrial and commercial users to collect electricity consumption. Smart meter using RS485 as its communication protocol collects data about electricity usage at a sampling interval of 15 minutes. Then, the collected data is transmitted via the RS485 protocol. To connect the smart meters to routers, an RS485 to RJ45 converter is used, converting the differential signals of RS485 to the voltage levels used by Ethernet. The router then receives the collected data and forwards it to the cloud system over a standard internet connection. For weather stations, *m* sensors are installed in three locations respectively and meteorological data is collected from 2015. Each sensor is responsible for collecting different meteorological characteristics such as temperature, humidity, and rainfall. Similarly, these sensors using RS485 as their communication protocol collect data about weather conditions at a sampling interval of 15 minutes. Then, the collected data is transmitted via the RS485 protocol. To connect the smart meters to routers, an RS485 to RJ45 converter is also used, converting the differential signals of RS485 to the voltage levels used by Ethernet. The router then transmits the meteorological data collected by these sensors to the cloud system through the Internet. After the cloud system receives the collected data, it transmits the data to the cloud database through the Internet for storage. Figure 1 illustrates the proposed communication platform.

### Measurement of selected users

386 users from three cities in southern China are chosen for measurement, and smart meters are used to collect 15-minute sampling electricity consumption data. These users cover 17 sections and 45 divisions, according to the International Standard Industrial Classification of All Economic Activities (ISIC)^18^. These users are all industrial or commercial electricity customers, such as factories, retailers, enterprises, and public organizations. To maintain information security, the user names and locations are not disclosed in this paper. The detailed categories and corresponding numbers of users are listed in Table 1 (Production-related sections) and Table 2 (Services-related sections).

**Table 1:** The classifications of users (Production-related).

User Categories				
Section Code	Section Name	Division Code	Division Name	Number
A	Agriculture, forestry and fishing	01	Crop and animal production, hunting and related service activities	4
		02	Forestry and logging	6
C	Manufacturing	10	Manufacture of food products	8
		11	Manufacture of beverages	2
		13	Manufacture of textiles	22
		14	Manufacture of wearing apparel	7
		15	Manufacture of leather and related products	2
		16	Manufacture of wood and of products of wood and cork, except furniture; manufacture of articles of straw and plaiting materials	2
		17	Manufacture of paper and paper products	8
		18	Printing and reproduction of recorded media	5
		20	Manufacture of chemicals and chemical products	1
		21	Manufacture of pharmaceuticals, medicinal chemical and botanical products	1
		22	Manufacture of rubber and plastics products	31
		23	Manufacture of other non-metallic mineral products	34
		24	Manufacture of basic metals	23
		25	Manufacture of fabricated metal products, except machinery and equipment	45
		26	Manufacture of computer, electronic and optical products	11
		27	Manufacture of electrical equipment	1
		28	Manufacture of machinery and equipment n.e.c.	12
		29	Manufacture of motor vehicles, trailers and semi-trailers	5
		31	Manufacture of furniture	18
		32	Other manufacturing	6
		33	Repair and installation of machinery and equipment	1
D	Electricity, gas, steam and air conditioning supply	35	Electricity, gas, steam and air conditioning supply	1
E	Water supply; sewerage, waste management and remediation activities	37	Sewerage	3
F	Construction	41	Construction of buildings	1
		42	Civil engineering	2
		43	Specialized construction activities	1

**Table 2:** The classifications of users (Services-related).

User Categories				
Section Code	Section Name	Division Code	Division Name	Number
G	Wholesale and retail trade; repair of motor vehicles and motorcycles	45	Wholesale and retail trade and repair of motor vehicles and motorcycles	7
H	Transportation and storage	52	Warehousing and support activities for transportation	1
		53	Postal and courier activities	2
I	Accommodation and food service activities	55	Accommodation	2
		56	Food and beverage service activities	4
J	Information and communication	62	Computer programming, consultancy and related activities	1
K	Financial and insurance activities	64	Financial service activities, except insurance and pension funding	17
		66	Activities auxiliary to financial service and insurance activities	1
L	Real estate activities	68	Real estate activities	54
M	Professional, scientific and technical activities	71	Architectural and engineering activities; technical testing and analysis	1
N	Administrative and support service activities	80	Security and investigation activities	1
		82	Office administrative, office support and other business support activities	3
O	Public administration and defence; compulsory social security	84	Public administration and defence; compulsory social security	17
P	Education	85	Education	6
Q	Human health and social work activities	86	Human health activities	2
		87	Residential care activities	2
S	Other service activities	96	Other personal service activities	2

### Data processing and generation

After data is collected from smart meters and sensors, data preprocessing is required to deal with missing data, outliers, and frequency inconsistencies. Next, methods for data processing and data generation are introduced.

#### Data preprocessing

After smart meter data is recorded and collected, differential operations are first conducted to obtain the electricity consumption data within the time period. Then data cleaning is performed on missing data, duplicated data and outliers. Missing values are filled with the last valid observation forward. Duplicated data is removed except for the first occurrence. Outliers are first detected based on *Z*-score^19^ which is shown in Eq. (1). *x* is the data, *μ* is the mean value of *x* and *σ* is the standard deviation of *x*. *Z* is set to three. The detected outliers are replaced with the mean value of the previous two observations. For data points not following a standard normal distribution, outliers are then detected using Interquartile Range (IQR) method^20^ and are replaced with the mean value of the previous 96 observations. Finally, the high-quality electricity consumption data of 386 users are obtained at a sampling interval of 15 minutes.

1 $$Z=\frac{x-\mu }{\sigma }$$

For the meteorological data collected by weather stations, data cleaning is also carried out in a similar manner as above. Duplicated meteorological data is first removed, and missing data is filled with the last valid observation. Based on the IQR method, outliers candidate sets are determined. If the value in the candidate set is not within the two times *Z*-score intervals of the previous two observations, the value will be replaced with the mean value of the previous two observations. The original meteorological data has a sampling interval of 30 minutes. To keep it consistent with the frequency of the load data for further analysis, linear interpolation is used to upsample it to obtain meteorological data with a sampling interval of 15 minutes^21^.

#### Selected extreme weather events

Studying the impact of extreme weather on electricity consumption patterns is very important for power supply security. There are 20 types of extreme weather events including high temperatures, high humidity, tropical storm, typhoon, and heavy rain are selected in this paper. The three cities where users in the dataset are all located in southern China and have a subtropical humid climate. Winters are short, mild, and relatively dry, while summers are long, hot, and very wet. The criteria of extreme weather events are defined based on statistical data, national standards, etc., combined with the characteristics of the three cities. The detailed descriptions of extreme weather events and their criteria are shown in Table 3. For EW1 to EW4, four extreme weather events are defined based on statistical values of temperature and humidity. For EW5 to EW7, three thunderstorm weather events are defined based on the wind gust data, according to the wind threat definition by National Oceanic and Atmospheric Administration (NOAA)^22^. For EW8 to EW12, some tropical storm and typhoon weather events are defined based on wind speed observations according to the Beaufort wind force scale. Moreover, typhoon data that occurred within a radius of 500 kilometers of these three cities from 2015 to 2022 is additionally collected as a supplement, including the typhoon name, time, and intensity. For EW13 to EW20, some other extreme weather events are identified based on the weather condition data.

**Table 3:** The classifications of extreme weather.

No.	Extreme Weather	Criterion
EW1	Low temperature	Temperature below lower bounds of the 95% confidence interval of temperatures between 2015 to 2022 in the city, e.g, 50°F (Fahrenheit) for City 1
EW2	High temperature	Temperature above upper bounds of the 95% confidence interval of temperatures between 2015 to 2022 in the city, e.g, 95°F for City 1
EW3	High humidity	Relative humidity(%) above upper bounds of the 95% confidence interval of humidity between 2015 to 2022 in the city, e.g, 97.85% for City 1
EW4	High heat and humidity	Temperature larger than 95°F and relative humidity larger than 60%
EW5	Severe thunderstorm - Damaging Wind Gusts	Wind gust larger than 58 mph and smaller than 74 mph (miles per hour)
EW6	Severe thunderstorm -Very Damaging Wind Gusts	Wind gust larger than 74 mph and smaller than 91 mph
EW7	Severe thunderstorm -Violent Wind Gusts	Wind gust larger than 91 mph
EW8	Tropical Storm	Wind speed larger than 39 mph and smaller than 54 mph
EW9	Severe Tropical Storm	Wind speed larger than 54 mph and smaller than 73 mph
EW10	Typhoon	Wind speed larger than 73 mph and smaller than 93 mph
EW11	Strong Typhoon	Wind speed larger than 93 mph and smaller than 114 mph
EW12	Super Typhoon	Wind speed larger than 114 mph
EW13	Heavy Rain	Weather condition equals ‘Heavy Rain’
EW14	Heavy Rain/Windy	Weather condition equals ‘Heavy Rain/Windy’
EW15	Heavy Rain Shower	Weather condition equals ‘Heavy Rain Shower’
EW16	Heavy Rain Shower/Windy	Weather condition equals ‘Heavy Rain Shower/Windy’
EW17	Heavy T-Storm	Weather condition equals ‘Heavy T-Storm’
EW18	Heavy T-Storm/Windy	Weather condition equals ‘Heavy T-Storm/Windy’
EW19	Light Sleet	Weather condition equals ‘Light Sleet’
EW20	Light Sleet/Windy	Weather condition equals ‘Light Sleet/Windy’

Following these steps, a large-scale industrial and commercial load dataset in extreme weather events is constructed, named Extreme Weather Events Load Dataset (EWELD). It includes electricity consumption data of industrial and commercial users under extreme weather events such as typhoons and extreme heat, which are collected at 15-minute intervals. The data is collected over six years from smart meters installed at the power entry points of users in southern China. The dataset consists of electricity consumption data from 386 industrial and commercial users in 17 industries, with more than 50 million records. During the recording period, extreme weather events such as typhoons and extreme heat are marked to form a total of 5,741 event records.

## Data Records

EWELD is available to the public through Figshare^23^ in .csv or .xlsx formats. Table 4 provides a summary of the folder structures, description of data files, output variables in each file, and the format of these files. The whole dataset includes four folders, among which the electricity consumption and weather data folders contain the basic data, extreme weather and user location folders contain the necessary supplementary information, including extreme weather filtering criteria and other useful indexes.

**Table 4:** Description of EWELD.

Folder	Files Description	Data Description*
Electricity Consumption	386 .csv files represent the 15-minute sampling electricity consumption data (Unit: kWh) for different users, named U1, U2,…U386 following the order of industrial classification. These files are organized into 17 sections folders and 45 divisions subfolders, as shown in Tables 1, 2. E.g., subfolder A01 has four files, named U1, U2, U3, U4.	Data Dimension: variable (R) 2 (C) C1: Time, C2: Value
Weather Data	Three .csv files represent the 15-minute sampling meteorological characteristics data for 3 cities from 2015/01/01 00:00 to 2022/11/01 23:30, named W1, W2, W3.	Data Dimension: 274751 (R) 9 (C), C1: Time, C2: Temperature(F), C3: Dew Point(F), C4: Humidity(%), C5: Wind, C6: Wind Speed(mph), C7: Wind Gust(mph), C8: Pressure(in), C9: Condition
	A .xlsx file represents the typhoon data which contains the tropical storm and typhoon events (EW8 to EW12) that have occurred within 500 km of the 3 cities from 2015/01/01 to 2022/11/2	Data Dimension: 61 (R) 4 (C) C1: NO., C2: Start Time, C3: End Time, C4: Weather
Extreme Weather	A .xlsx file represents the filtering criteria for various extreme weather events, same as Table 3	Data Dimension: 20 (R) 3 (C) C1: NO., C2: Extreme Weather, C3: Criterion
	Three folders represent the filtered time interval of extreme weather events for three cities are contained, named EW CT1, EW CT2, and EW CT3. For each folder, 20 .csv files are contained, which represent the information on extreme weather events.	Data Dimension: variable (R) 4 (C) C1:Time, C2:Start Time, C3:End Time, C4:Weather
	A .csv file counts the number of different types of extreme weather in three cities these years, named ‘count_20class_interval_df’.	Data Dimension: 20 (R) 4 (C) C1:EW, C2:CT1, C3:CT2, C4:CT3
User Location	Three .csv files represent the users’ information in 3 cities, named U CT1, U CT2, U CT3.	Data Dimension: variable (R) 1 (C) C1: User NO.

*Note: In ‘Data File Description’, C = column, R = Row. C[*i*] indicates the *i*-th column of a data file.

For the electricity consumption data of 386 users, some features are summarized in Table 5. The electricity consumption data covers from June 2016 to August 2022, with multiple variable records for different users. The statistics of the electricity consumption data for different types of users are shown in Table 6, including mean values, standard deviation, skew, kurtosis, and percentiles. Electricity consumption of manufacturing users (Section C) has a large standard deviation since there are various types and sizes of manufacturing factories. Users of Section C and G tend to operate with higher power consumption, and users of Section S (Other service activities) consume lower electricity. There are 274,752 weather data records with a 15-minute sampling interval for each city, covering the period from 01/01/2015 00:00 to 01/11/2022 23:30. The weather data statistics for different numerical meteorological indicators in three cities are shown in Table 7.

**Table 5:** Summary of users’ electricity consumption data.

File No.	Characteristics	Industry Section	Location city	Start Time	End Time	Records	Frequency (Hz)
U1-U386	Overall	A-S	CT1, CT2, CT3	02/06/2016 00:15	10/08/2022 23:45	386 files	1/900
U57	Maximum time range	C17	CT2	02/06/2016 00:15	09/08/2022 23:45	216959	1/900
U189	Minimum time range	C25	CT2	02/08/2017 00:15	08/08/2017 00:00	576	1/900
U157	Maximum power consumption	C25	CT2	02/01/2018 09:15	25/10/2021 15:30	133658	1/900
U243	Minimum power consumption	C31	CT2	02/03/2018 16:15	17/04/2021 10:00	109608	1/900
U108	Maximum mean electricity consumption	C23	CT2	24/05/2018 00:15	25/10/2021 15:00	120060	1/900
U106	Minimum mean electricity consumption	C23	CT2	19/04/2018 17:45	11/05/2022 11:45	142345	1/900

**Table 6:** Summary of users’ electricity consumption data statistics.

Industrial Section	Mean	Standard deviation	Skew	Kurtosis	0^*th*^ percentile	2.5^*th*^ percentile	50^*th*^ percentile	97.5^*th*^ percentile	100^*th*^ percentile
A	37.36	72.68	3.32	11.49	0	0	8.68	314.27	444.46
C	77.75	253.21	8.46	104.29	0	0	10.52	598.96	7977.2
D	16.3	11.03	0.39	−0.32	0	0.13	16.1	39.66	53.45
E	49.96	91.12	2.17	3.59	0	0	14.6	331.09	377.66
F	11	28.83	4.25	18.89	0	0	1.87	124.5	214.27
G	79.68	160.05	3.99	18.51	0	0	29.49	589.58	1280.84
H	14.67	17.09	1.3	0.97	0	0	7.3	59.19	82
I	51.32	67.33	1.73	2.17	0	0	21.9	243.12	341.75
J	3.9	2.86	2.77	9.57	0	1.06	3.18	13.23	21.7
K	21.92	37.61	2.88	8.85	0	0	7.51	159.26	219.69
L	36.29	60.24	2.97	11.52	0	0	12.44	208.44	546.44
M	8.68	12.98	1.79	1.95	0	0.81	2.09	45.54	52.77
N	22.17	23.88	1.89	4.99	0	0	17.97	94.19	151.54
O	20.79	33.93	3.55	16.25	0	0	8.39	119.69	299.27
P	24.24	58.45	4.99	30.9	0	0	3.03	193.8	631.64
Q	25.54	33.89	2.89	12.34	0	0	14.91	122.41	283.94
S	17.53	23.26	1.06	−0.3	0	0	1.77	70.1	86.84

**Table 7:** Summary of weather data statistics.

City	Meteorology	Mean	Standard deviation	skew	kurtosis	minimum	2.5^*th*^ percentile	50^*th*^ percentile	97.5^*th*^ percentile	maximum
C1	Temperature	74.91	11.86	−0.43	−0.46	35.58	50.14	77.08	94.46	105.28
	Dew Point	63.85	13.36	−0.95	0.43	0.98	32.82	67.33	79.86	85.4
	Humidity	70.75	17.27	−0.57	−0.15	11.96	31.55	73.02	97.15	102
	Wind Speed	6.15	3.96	1.07	1.33	0	1	4.59	16.0	38.45
	Wind Gust	0.18	1.97	13.03	195.39	0	0	0	0	53.61
	Pressure	29.85	0.37	0.04	−0.46	28.55	29.16	29.84	30.55	31.25
C2	Temperature	74.38	10.01	−0.57	−0.2	29.92	52.88	76.42	89.93	98.81
	Dew Point	66.75	11.6	−1.07	0.99	12.03	39.2	69.66	80.06	92.25
	Humidity	78.53	14.01	−0.79	0.43	20.97	44.87	80.84	98.92	101.96
	Wind Speed	7.84	3.9	0.89	2.82	0	1.97	7.5	16.64	58.06
	Wind Gust	0.14	1.72	15.63	298.13	0	0	0	0	65.89
	Pressure	29.78	0.34	0.01	−0.47	28.21	29.14	29.78	30.43	30.89
C3	Temperature	75.12	9.67	−0.51	−0.58	38.3	54.76	76.97	89.27	100.93
	Dew Point	68.73	10.89	−0.86	0.16	26.97	44.3	71.56	81.88	87.47
	Humidity	81.01	11.24	−0.98	1.11	34.3	52.24	82.99	98.08	102
	Wind Speed	10.65	5.37	0.76	1.66	0	1.99	9.91	22.7	72.93
	Wind Gust	0.12	2.06	23.27	656.52	0	0	0	0	95.38
	Pressure	29.87	0.36	0.01	−0.46	28.18	29.2	29.87	30.55	31.11

## Technical Validation

The sensors and data acquisition devices are manufactured by well-known companies that produce meters and sensors for industrial and residential installations around the world. Meter calibration is done by the meter manufacturers before shipping to the factory. The calibration process is proprietary, and we are not privy to the process. Additionally, we checked if the measured values are within the range given by the manufacturers of the meters. Measurements outside the range or at its edge suggest a wrongly designed measurement infrastructure. All measurements are found to be well within the given range though. This section presents the data visualization to show the quality and technical validity of the dataset, including data integrity, reliability, and effectiveness. The data integrity is presented by data availability plots. The data reliability is verified by determining the discrepancy of annual total electricity consumption of these users of two metering systems. In addition, annual, seasonal, monthly, weekly, and daily pattern plots are represented to provide characteristic insights into electricity consumption and operating states of different industries. The data effectiveness is then verified by electricity consumption diagrams around extreme weather events to demonstrate the impact of extreme weather on electricity consumption.

### Data integrity

**Fig. 2:**
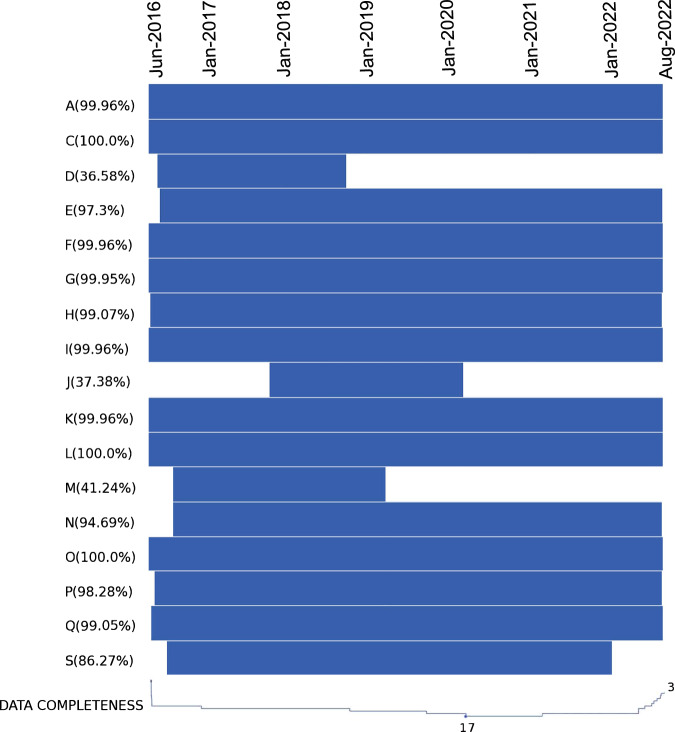
Data availability for all industrial sections from June 2016 to August 2022 (blue = data available).

EWELD mainly includes two types of data, electricity consumption data of 386 users and weather data from 3 cities where these users are located. After data cleaning, the weather data realizes approximately 100% data availability from January 2015 to October 2022. The electricity consumption data comprises 217,055 15-minute steps and shows various availability for the data collection period (02/06/2016 ~ 10/08/2022) due to some force majeure factors. These factors include but are not limited to the different installation time points of smart meters, closures of some factories, communication network interruptions, and data collection failures. Among 386 users, there are 30 users with data completeness greater than 99% and 63 users greater than 90%. For a better overview, data availability for each industrial section is visualized in Fig. 2. The available data plot for the entire data collection period (where the missing data is indicated using white lines) is shown on the left side of the figure. Most industrial sections have large data availability with three sections less than 50%, D (Electricity, gas, steam and air conditioning supply), J (information and communication), M (Professional, scientific and technical activities). Only one user is collected for each of these three industrial sections (U256, U280, U353). These users only have data available for some time for various reasons. For example, we started to collect smart meter data of U256 from customers in July 2016 but stopped collecting due to the user changed their business venue in November 2018. Note that data missing is mainly caused by data gaps larger than one day. The completeness criterion states that a complete day has at least 95% of the expected records^24^. Statistical results indicate that more than 99% of the days have more than 91 records, i.e. the vast majority of days meet the completeness criterion. Moreover, missing data can be further recovered through missing data imputation methods to guarantee data quality, such as regression-based and deep-learning-based methods^25,26^.

### Data reliability

**Fig. 3:**
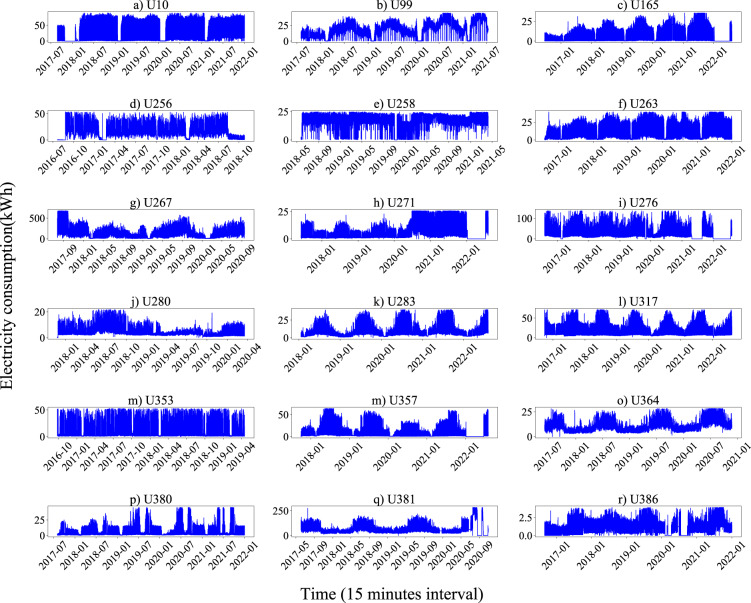
Annual electricity consumption profiles of 18 users from various industries during data collection periods. (**a**) U10 (A02 Forestry and logging); (**b**) U99 (C22 Manufacture of rubber and plastics products); (**c**) U165 (C25 Manufacture of fabricated metal products, except machinery and equipment); (**d**) U256 (D35 Electricity, gas, steam and air conditioning supply); (**e**) U258 (E37 Sewerage); (**f**) U263 (F43 Specialized construction activities); (**g**) U267 (G45 Wholesale and retail trade and repair of motor vehicles and motorcycles); (**h**) U271 (H52 Warehousing and support activities for transportation); (**i**) U276 (I56 Food and beverage service activities); (**j**) U280 (J62 Computer programming, consultancy and related activities); (**k**) U283 (K64 Financial service activities, except insurance and pension funding); (**l**) U317 (L68 Real estate activities); (**m**) U353 (M71 Architectural and engineering activities, technical testing and analysis); (**n**) U357 (N82 Office administrative, office support and other business support activities); (**o**) U364 (O84 Public administration and defence; compulsory social security); (**p**) U380 (P85 Education); (**q**) U381 (Q86 Human health activities); (**r**) U386 (S96 Other personal service activities).

**Fig. 4:**
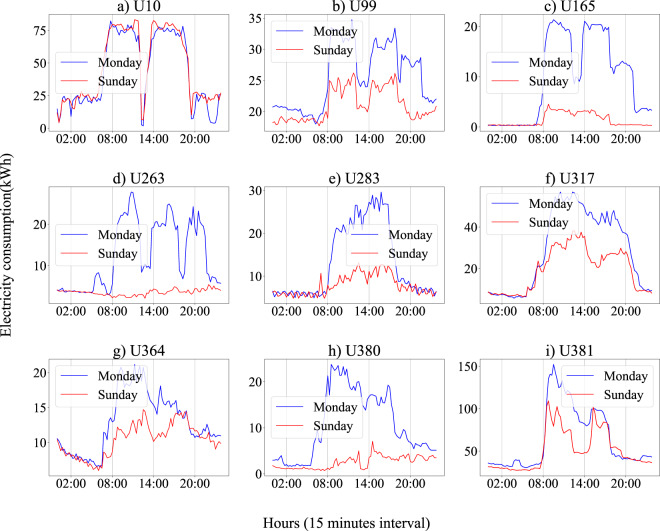
Monthly electricity consumption patterns of 9 users.

**Fig. 5:**
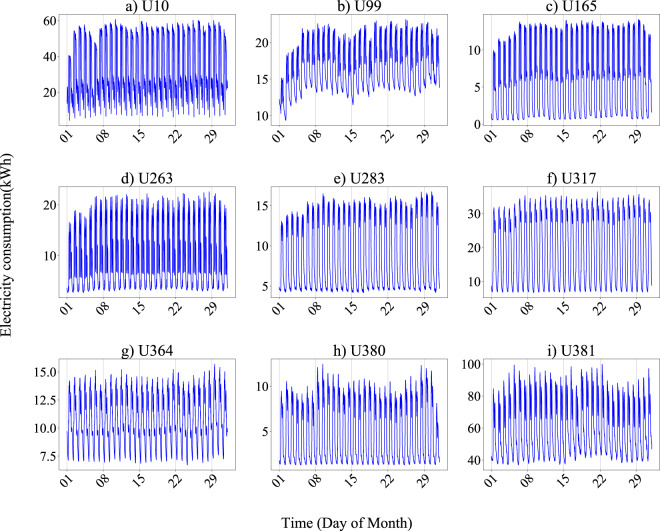
Weekly electricity consumption patterns of 9 users.

**Fig. 6:**
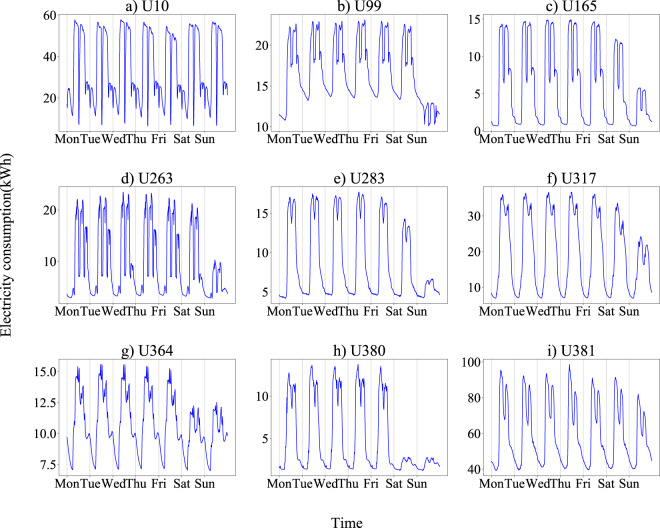
Daily electricity consumption profiles of 9 users on weekday (Monday) and weekend (Sunday).

The annual total electricity consumption according to the dataset is compared with the value reported by the electric company. Very close values are obtained with errors less than 2%. For further validation, the annual, monthly, weekly, and daily pattern plots of some users are presented to illustrate the quality of records properly. The energy consumption discretized in 15-minute intervals for 18 representative users during the data collection period is presented in Fig. 3, covering 17 industrial sections. It shows an overview of the electricity consumption of various users these years. Different types of users show various electricity consumption characteristics. For instance, U165 (Steel pipe factory) uses more electricity in the mid-year and shows a typical single-peak electricity consumption curve throughout the year, while U258 (sewer systems) presents no significant seasonal variations in electricity consumption all year round. Next, monthly, weekly, and daily statistics of users are drawn to show the significant consumption patterns, aligning with the corresponding electricity consumption behavior of different industries and verifying the reliability of this dataset. Fig. 4 illustrates the monthly electricity consumption profiles of 9 users from different industrial sections, obtained by averaging the electricity consumption during the data collection period by day of the month. All users show a regular periodical change. Fig. 5 shows the weekly electricity consumption patterns, obtained by averaging the electricity consumption during the data collection period by day of the week. Daily electricity consumption patterns are shown in Fig. 6, with weekday and weekend differences for distinct users. U10 (timber production company), U364 (police station), and U381 (hospital) show little difference in electricity consumption on Monday and Sunday. At the same time, the other six users present lower electricity consumption on Sunday than Monday, especially for U165 (steel pipe factory) and U380 (primary school).

### Data effectiveness

**Fig. 7:**
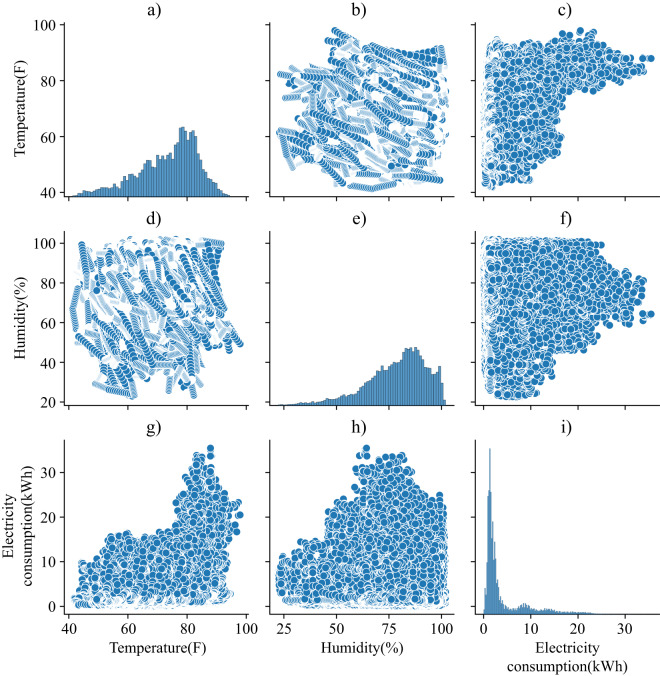
The relationship between electricity consumption and weather indications (temperature and humidity) of U380 in 2018.

To show the effectiveness of the dataset in capturing dynamic changes and reflecting the impact of significant events on electricity consumption, U380 is chosen as an example to analyze the weather impact on its electricity consumption. U380 is a primary school located in City C2. Fig. 7 presents the relationship between electricity consumption and two important meteorological indicators, temperature and humidity, in 2018. The diagonal subplots show the distribution of values for different variables. For example, temperature data presents a Poisson Distribution in Fig. 7a. Non-diagonal subplots show the scatter plots between two variables and it can be found that the electricity consumption of U380 has a strong correlation with temperature and humidity, aligning with the law of facts. Specifically, higher temperatures and humidity tend to result in higher electricity consumption, which is caused by greater dependency on air conditioners and electric fans. Taking Fig. 7g as an example, temperature and electricity consumption are positively related. The chaotic points in Fig. 7d indicate that there is no evident relationship between temperature and humidity.

**Fig. 8:**
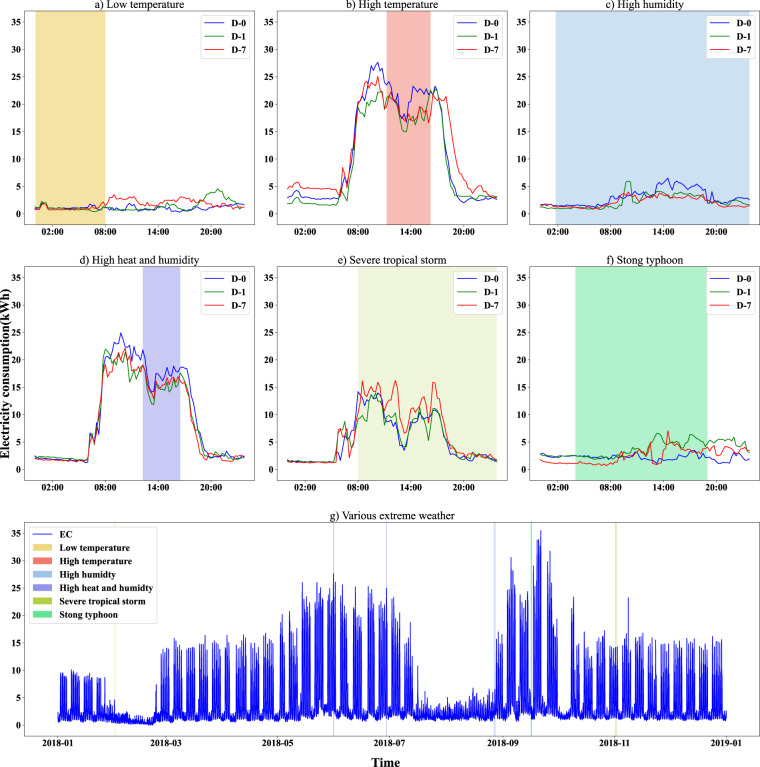
Impacts of various extreme weather events on the electricity consumption of U380 in 2018. (**a**) Low temperature; (**b**)High temperature; (**c**) High humidity; (**d**) High heat and humidity; (**e**) Severe tropical storm; (**f**) Strong typhoon; (**g**) The time of different types of extreme weather in 2018. Shaded areas show the period of different extreme weather events. Different color lines represent daily electricity consumption curves of the different days: the day extreme weather happened (D-0) in the blue line, the previous day in the green line, and the same day of the last week in the red line.

Next, the impact of different types of extreme weather on its electricity consumption is shown in Fig. 8, indicating the consistency with the reality that various extreme events lead to different impacts on electricity consumption. It presents the electricity consumption of the day extreme weather happened (D-0) and compares it with that of the previous day (D-0) and the same day of the last week (D-7). Low temperature has little influence on electricity consumption since some of the central air conditioners in these three cities do not have the heating capability, and the lowest temperatures in these cities in 2018 are still above 41 Fahrenheit degrees without the need to turn on the air conditioners for heating. On the other hand, high temperatures and high humidity will lead to higher electricity consumption due to air conditioning cooling and dehumidification. In addition, tropical storms and typhoons tend to result in lower electricity consumption since the school generally chooses to be closed to ensure safety after receiving the warning weather information.

## Usage Notes

For the privacy protection of the users, the raw data collected is not publicly available. Although the EWELD dataset published here is the data preprocessed through some simple data cleaning procedures, significant information remained unchanged, such as the electricity consumption patterns in different cycles. The codes published are licensed under the MIT license. We recommend users follow the guidance on Github, including the details of installation, package usage, dataset navigation, and code navigation. The raw data for this project was obtained through a collaboration between the authors of this work and an industry partner. This partner is a well-respected power retailer and was integral in supplying the information we needed for our analyses. Unfortunately, while we are unable to share the exact source due to confidentiality agreements, researchers who wish to repeat our work or perform similar studies can approach such companies. This could be either through direct contact or by establishing collaborations as we did for this project. Please note that obtaining such data might require appropriate agreements regarding data privacy and usage to be put in place. We recommend researchers consider such ethical aspects when planning their projects.

The dataset can significantly contribute to multiple potential applications. Firstly, in user behavior analysis, the dataset can reveal valuable insights into patterns and habits of energy consumption. This can aid in the development of targeted energy conservation strategies, thereby optimizing the power system for user-specific demands. Secondly, the dataset can contribute greatly to load forecasting. Accurate load forecasting is essential in maintaining the balance between energy supply and demand, hence avoiding possible outages and inefficiencies. The rich data available can enhance the precision of short-term and medium-term load predictions, assisting utility companies in making more informed decisions about resource allocation and grid management. Lastly, the dataset can facilitate the analysis of extreme event impacts. Understanding how extreme weather events affect power loads is crucial in this era of growing climate uncertainties. This dataset allows scholars and policymakers to quantify and predict these impacts, leading to the development of more resilient power systems that can withstand such events. Each of these applications holds promise for improving the efficiency, resilience, and sustainability of our power systems.

## Data Availability

The code implementation was done using Python. Source codes that were used to develop and analyze the data are publicly available in the GitHub repository (https://github.com/Judy0718/EWELD).
